# Ultrasound-assisted enzymatic extraction of dark tea total polyphenols

**DOI:** 10.3389/fnut.2025.1548103

**Published:** 2025-06-27

**Authors:** Jinhua Shao, Xinsheng Xiao, Yuhang Li, Bo Chen, Lei Xing, Zhiyong Zhu, Chengmei Qi

**Affiliations:** School of Chemistry and Bioengineering, Hunan University of Science and Engineering, Yongzhou, China

**Keywords:** dark tea, ultrasound-assisted enzymatic method, polyphenols, antioxidant activity, response surface methodology

## Abstract

Tea polyphenols have become the most biologically active components in tea, and they are also one of the key factors determining the color, flavor, health benefits. This study used dark tea as the raw material and employed the Plackett Burman method to screen for 8 factors that may affect the extraction of dark tea total polyphenols. Box Behnken response surface methodology was used to further optimize the four most important variables. At a solid-liquid ratio of 1:50, enzyme dosage of 2.5%, pH of 5.6, enzymatic hydrolysis temperature of 45°C, enzymatic hydrolysis time of 50 min, ethanol volume fraction of 50%, ultrasonic temperature of 72°C, and ultrasonic time of 50 min, the highest extraction amount of dark tea total polyphenols was obtained. Compared with the ethanol reflux extraction method, the extraction amount of dark tea total polyphenols increased by about 43.38%, and compared with the ultrasonic assisted extraction method, the extraction amount of dark tea total polyphenols increased by about 30.45%. Ultrasonic assisted enzymatic extraction of dark tea total polyphenols has the strongest antioxidant activity. The optimized process of ultrasound assisted enzymatic extraction can increase the extraction amount and antioxidant activity of dark tea total polyphenols, reduce extraction time, and lower extraction temperature. Ultrasonic assisted enzymatic method is simple, efficient, and can be industrially promoted, and it also has reference value for the extraction of other plant active ingredients.

## 1 Introduction

Tea is consumed throughout the world and is rich in active ingredients such as polyphenols, catechins, theaflavins, theanine, polysaccharides, and caffeine. Tea can not only be used as a beverage, but also utilized in the preparation of ice cream, candies, biscuits, sauces, and other products to enhance its nutritional value (1, 2). Dark tea is one of the six major tea categories and describes a form of tea that is fermented by microorganisms, leading to the accumulation of high concentrations of active substances (3). Tea polyphenols have become the most biologically active components in tea, and they are also one of the key factors determining the color, flavor, health benefits, and pharmacological properties of tea (4), accounting for 20%−30% of the dry weight of tea and acting as natural antioxidants. In addition, tea polyphenols have been shown to be effective in reducing fatigue, anti-aging effects, and lowering of blood sugar and lipids, as well as having anti-cancer, anti-inflammatory, antiviral, and anti-obesity properties and promoting muscle health (5, 6).

Enzymatic hydrolysis of plant cells breaks down the cell wall structure of the cells, allowing the release of active ingredients and reducing the extraction time (7). Enzyme-assisted extraction method offers several advantages, including high selectivity, rapid reaction speed, mild reaction conditions, and high extraction efficiency (8, 9). Ultrasound-assisted extraction (UAE) has the advantages of being cost-effective with lower equipment requirements, less solvent usage, low temperature, and short extraction time, all of which are useful for the extraction of thermo-labile compounds (10, 11). Ultrasound-assisted enzyme extraction is an emerging extraction technology in the pharmaceutical and food industries (12, 13), offering advantages such as high extraction efficiency, low energy consumption, minimal solvent usage, reduced environmental pollution, and simple operation (14, 15). This method has been employed to extract polyphenols from various other plant materials (16, 17). In cases where extraction rates using a single method are low, therefore, ultrasound-assisted enzymatic extraction may be effective, such as in the extraction of dark tea total polyphenols.

The response surface method (RSM) is an optimization method that integrates experimental design and mathematical modeling and can reduce the number of experiments, examine interactions between influencing factors, and reduce experimental time and cost (18). Therefore, this study first identified the key variables associated with the extraction of total polyphenols from dark tea using the Plackett-Burman experimental design, followed by the Box-Behnken test to determine the optimal process conditions. At the same time, the effects of conventional ethanol reflux extraction method, ultrasound assisted extraction method, and ultrasound assisted enzyme method on the extraction amount and antioxidant activity of dark tea total polyphenols were compared. LC-MS/MS chromatography was used to qualitatively and quantitatively analyze the differences in phenolic substance extraction under different extraction conditions, in order to provide scientific reference for the better development of dark tea total polyphenols. Ultrasound-assisted enzymatic extraction methods integrate traditional and modern techniques, overcoming some of the limitations of conventional methods. Compared to other techniques, ultrasound-assisted enzymatic extraction stands out as an emerging, efficient method capable of synergistically degrading plant cell walls to accelerate polyphenol release. Additionally, research on polyphenolic substances in dark tea from Yongzhou, Hunan Province, China, has not been previously reported.

## 2 Materials and methods

### 2.1 Preprocessing

The dark tea used was the gold award small bag tea from the Hunan Ziranyun Dark Tea Technology Co., Ltd. (Qiyang City, Hunan Province, China). The tea bags were placed in an oven at 50°C to dry to constant weight. The tea leaves were then removed and ground using a grinder, passed through an 80-mesh sieve, and stored in sealed bags for later use.

### 2.2 Ultrasound-assisted enzymatic extraction of dark tea total polyphenols

The extraction process of dark tea total polyphenols was optimized in three steps. First, a single-factor test was performed, after which the Plackett-Burman method was used to identify the key factors in the extraction process using the results of the single-factor test. The identified key factors were then further optimized using the Box-Behnken test. As previously described (19), the Folin-Ciocalteu colorimetric method was used to measure the total polyphenols content, using a Model 752 UV-Vis spectrophotometer manufactured by Shanghai Sunny Hengping Scientific Instrument Co., Ltd. A linear regression was performed with the mass concentration of gallic acid standard (X, mg/ml) as the independent variable and absorbance (Y) as the dependent variable, yielding the regression equation: *Y* = 0.00924X + 0.03443(*R*^2^ = 0.9997), with a concentration range of 0–70 μg/ml. The amount of extracted total polyphenols was calculated according to the Equation 1:


(1) $$\begin{array}{l} \text{Extraction amount of total polyphenols }\left(\text{mg}/\text{g}\right)=\frac{\text{cVn}}{1000w} \end{array}$$

Where c represents the concentration of tea total polyphenols obtained from the regression equation (μg/ml), *V* indicates the volume of the extraction liquid (ml), *n* represents the dilution ratio, and w indicates the weight of the tea leaves (g).

#### 2.2.1 Single factor test

1.0 g of dark tea powder was weighed and placed into a round-bottom flask along with a specified ratio of water. A certain amount of cellulase (activity unit: 50,000 U/g) was added, and the pH was adjusted to an appropriate level. After enzyme hydrolysis at a set temperature for a defined time, the mixture was filtered through 8 layers of gauze to remove solid particles and collect the first filtrate. The first filtrate was then subjected to vacuum filtration to collect the second filtrate. The total polyphenol content in the second filtrate was determined using the Folin-Ciocalteu colorimetric method. Each experiment was repeated three times. The fixed solid-to-liquid ratio was set at 1:30 g/ml, with cellulase added at 2.5%, pH set at 5.6, enzyme hydrolysis temperature at 50°C, and enzyme hydrolysis time at 40 min. The effects of various parameters on the extraction of total polyphenols from dark tea were investigated, including different solid-to-liquid ratios (1:10, 1:20, 1:30, 1:40, and 1:50 g/ml), enzyme addition (1.5, 2.0, 2.5, 3.0, and 3.5%), pH values (4.4, 5.0, 5.6, 6.2, and 6.8), enzyme hydrolysis temperatures (40, 45, 50, 55, and 60°C), and enzyme hydrolysis times (20, 30, 40, 50, and 60 min).

Fifty grams of dark tea powder were precisely weighed and added to a beaker with a specified amount of water, maintaining a solid-to-liquid ratio of 1:40. Cellulase was added at 2.5%, the pH was adjusted to 5.6, and the enzyme hydrolysis was carried out at 50°C for 50 min. After the enzyme hydrolysis under these conditions, an appropriate amount of anhydrous ethanol was added to the beaker, and the ethanol concentration was adjusted. The mixture was then subjected to ultrasonic extraction (with a fixed ultrasonic frequency of 40 kHz and fixed ultrasonic power of 240 W). After extraction at a set temperature and time, the mixture was filtered through 8 layers of gauze to remove solid matter, and the first filtrate was collected. The first filtrate was then subjected to vacuum filtration to collect the second filtrate. The total polyphenol content in the second filtrate was determined using the Folin-Ciocalteu colorimetric method. Each experiment was repeated three times. The ethanol concentration was fixed at 50%, the ultrasonic temperature was set to 65°C, and the ultrasonic extraction time was 40 min. The effects of various factors on the extraction of total polyphenols from dark tea were investigated, including different ethanol concentrations (30, 40, 50, 60, and 70%), different ultrasonic temperatures (45, 55, 65, 75, and 85°C), and varying ultrasonic extraction times (20, 30, 40, 50, and 60 min).

#### 2.2.2 Design of the Plackett-Burman experiment

Combined with the results of the single-factor test, the Plackett-Burman test is designed to identify key factors such as the solid-liquid ratio (a, g/ml), enzyme dosage (b, %), temperature of enzyme hydrolysis (c, °C), time of enzyme hydrolysis (d, min), pH(e), ethanol concentration (f, %), and ultrasonic temperature (g, °C), and time (h, min). The amount of extract was used as the response variable, and each factor was used at two levels, high and low. The factors and levels of the Plackett-Burman experiment are shown in Table 1.

**Table 1:** Factors and levels of Plackett-Burman experiment design for total polyphenols extraction.

**Variable**	**Level**	
	**Low (-1)**	**High (**+**1)**
a	1:30	1:50
b	2	3
c	45	55
d	40	60
e	5.0	6.2
f	50	70
g	55	75
h	40	60

#### 2.2.3 Design of the response surface optimization experiment

Based on the results of the Plackett-Burman test, the temperature of enzyme hydrolysis, solid-liquid ratio, ethanol concentration, and ultrasonic temperature were found to be key factors. Using the amount of extracted total polyphenols as the response variable, an optimization experiment with four factorsand three levels was designed using the Box-Behnken test principle, where A represents the temperature of enzyme hydrolysis (in °C), B represents the solid-to-liquid ratio (in g/ml), C represents the ethanol concentration (in %), and D represents the ultrasonic temperature (in °C). The factors and levels are shown in Table 2.

**Table 2:** Factors and levels of Box-Behnken response surface experiments for total polyphenols extraction.

**Level**	**Factor**			
	**A**	**B**	**C**	**D**
−1	45	1:30	50	55
0	50	1:40	60	65
1	55	1:50	70	75

### 2.3 Effects of different extraction methods on the amount of extracted total polyphenols

#### 2.3.1 Extraction of dark tea total polyphenols by the ethanol refluxing method

Fifty gram of dark tea powder was accurately weighed and placed in a beaker, after which 2,500 ml of 50% ethanol solution was added, and the mixed solution was poured into a round-bottomed flask. After the connection of the condenser tube, the mixture was extracted in a water bath by refluxing at 80°C for 50 min. The residue was then filtered through eight layers of gauze, and the filtrate was vacuum-dried (at a vacuum pressure of 50 kPa, a drying temperature of 80°C, for 48 h) and collected. The amount of total polyphenols extracted was measured using the Folin-Ciocalteu colorimetric method.

#### 2.3.2 Ultrasound assisted extraction of dark tea total polyphenols

We accurately weighed 50.0 g of dark tea powder and placed it in a beaker. We added 2,500 ml of 50% ethanol solution to it and poured the mixed solution into an ultrasonic extraction machine. The ultrasonic power was set to 360 W, with a frequency of 40 kHz. The extraction was carried out at 72°C for 50 min. After extraction, the filter residue was filtered off with 8 layers of gauze, and the filtrate was vacuum filtered and collected. The total polyphenols extraction amount was determined using the Folin Ciocalteu colorimetric method.

#### 2.3.3 Ultrasonic assisted enzymatic extraction of dark tea total polyphenols

Accurately weigh 50.0 g of dark tea powder and place it in a round bottom flask with a solid-liquid ratio (g/ml) of 1:50. Then add 2.5% cellulase to the round bottom flask, adjust the pH of the solution to 5.6, and perform condensation reflux enzymatic hydrolysis at 45°C for 50 min. Then pour the enzymatic hydrolysis solution into a suitable beaker, add a certain amount of anhydrous ethanol, adjust the ethanol concentration to 50%, and then put it into an ultrasonic extraction machine. The ultrasonic power is 360 W, with a frequency of 40 kHz. Ultrasonic treatment is carried out at 72°C for 50 min. After pouring out the extraction solution from the ultrasonic extraction machine, filter out the filter residue with 8 layers of gauze, vacuum filter the filtrate and collect it. The total polyphenols extraction amount is determined by Folin Ciocalteu colorimetric method.

### 2.4 Determination of the antioxidant activity of dark tea total polyphenols

#### 2.4.1 DPPH free radical clearance

The method in reference (20) has been slightly modified. Add 50 μl of dark tea total polyphenols extraction solutions with different mass concentrations (0.1, 0.2, 0.4, 0.6, 0.8, and 1.2 mg/ml) and vit. C (with concentrations of 0.1, 0.2, 0.4, 0.6, 0.8, and 1.2 mg/ml), solutions with different mass concentrations using different extraction methods (G:ethanol reflux extraction, H:ultrasound assisted extraction, and I:ultrasound assisted enzyme extraction) to a 96 well plate. Then add 50 μl of 0.2 mmol/L DPPH solution with an absorbance value of 0.552, shake well, and allow it to in the dark at room temperature for 30 min. Measure the absorbance *A*_*i*_ at 517 nm. Replace the DPPH solution with anhydrous ethanol solution to measure the absorbance *A*_*j*_ of the mixture of dark tea total polyphenols and ethanol. Replace the sample solution with anhydrous ethanol solution to measure the absorbance *A*_0_ of the mixture of ethanol and DPPH. Follow the steps below. The formula is used to calculate the DPPH radical scavenging rate.


(2) $$\begin{array}{l} DPPH\;free\;radical\;clearance\;rate\;\left(\%\right)\;=\;\frac{A_{0}-\left(A_{i}-A_{j}\right)}{A_{0}}\times 100 \end{array}$$

#### 2.4.2 Determination of ·OH free radical scavenging ability

Referring to the method in reference (21), with slight modifications. We transferred dark tea total polyphenols extraction solutions of different mass concentrations (0.1, 0.2, 0.4, 0.6, 0.8, and 1.2 mg/mL) using different extraction methods (G:ethanol reflux extraction, H:ultrasound assisted extraction, and I:ultrasound assisted enzyme extraction) into test tubes. We sequentially transferred 1 ml of 9 mmol/L FeSO_4_ solution and 1 ml of 9 mmol/L salicylic acid solution. After reacting at room temperature for 10 min, we immediately added 1 ml of 8.8 mmol/L H_2_O_2_ to terminate the reaction. After reacting in a 37°C water bath for 30 min, we measured the absorbance at 510 nm using a UV visible spectrophotometer, denoted as *A*_i_, and measured the absorbance using distilled water instead of H_2_O_2_, denoted as *A*_j_, and distilled water instead of the sample solution, denoted as *A*_j_. Take the same concentration of V_C_ solution as the positive control for *A*_0_. Take the average of three parallel measurements for each group. Calculate the hydroxyl radical scavenging ability according to Equation 3.


(3) $$\begin{array}{l} \text{OHfreeradicalclearancerate}\left(\text{\%}\right)=\frac{{\text{A}}_{0}-\left({\text{A}}_{\text{i}}-{\text{A}}_{\text{j}}\right)}{{\text{A}}_{0}}\times 100 \end{array}$$

### 2.5 LC-MS/MS analysis of dark tea total polyphenols

LC-MS/MS method was used to qualitatively and quantitatively analyze five polyphenolic compounds, namely gallic acid, chlorogenic acid, rutin, epicatechin, and catechin, in dark tea total polyphenols extracts extracted by different extraction methods.

#### 2.5.1 Sample preparation

Accurately weigh an appropriate amount of gallic acid, chlorogenic acid, rutin, epicatechin, and epicatechin reference standards and prepare a 5 μg/ml mixed standard solution using chromatographic methanol. Filter the solution through a 0.22 μm filter membrane and set it aside; Accurately weigh 10 mg of dark tea total polyphenols vacuum-dried powder (at a vacuum pressure of 50 kPa, a drying temperature of 80°C, for 48 h) using ethanol reflux extraction method, ultrasonic assisted extraction method, and ultrasonic assisted enzymatic method, dissolve it in chromatographic methanol and dilute to 10 ml, filter it through a 0.22 μm membrane, and set it aside.

#### 2.5.2 Chromatographic conditions

Chromatography column: ZOR BAX Extend C18 column, 3.5 μm, 4.6 mm × 50 mm (Agilent, USA); Mobile phase: E: 0.5% formic acid aqueous solution, F: 100% acetonitrile solution; The gradient elution method is shown in Table 3; Column temperature: 35°C; Flow rate: 0.2 ml/min; Injection volume: 10 μl.

**Table 3:** Gradient elution program.

**Time (min)**	***E* (%)**	***F* (%)**
0	95	5
1	95	5
8	75	25
12	40	60
13	0	100
16	0	100
16.1	95	5
20	95	5

#### 2.5.3 Mass spectrometry conditions

Ion source: electric spray ion source (ESI), scanning mode: positive and negative ion mode, scanning mode: multiple reaction monitoring (MRM), dry gas: N_2_, ion source temperature: 400°C, spray voltage (IS): 4.5–5.5 kv. The detection of ion parameters is shown in Table 4.

**Table 4:** Parameters for detecting ions.

**Components**	**Relative molecular mass**	**Ion pair**	**Ionic mode**	**Retention time**
Epicatechins	290.27	288.95 > 109.00	ESI^−^	11.149
chlorogenic acid	354.31	353.00 > 191.00	ESI^−^	10.136
gallic acid	170.12	168.90 > 124.90	ESI^−^	6.783
rutin	610.52	609.00 > 300.00	ESI^−^	12.816
Catechins	290.28	289.00 > 139.00	ESI^+^	11.822

### 2.6 Data processing

Excel 2007, IBM SPSS Statistics 27, Origin 2021, and Design Expert 11 software were used for data analysis and drawing.

## 3 Results and discussion

### 3.1 Effects of different factors on total polyphenols concentrations of extracts

**Figure 1:**
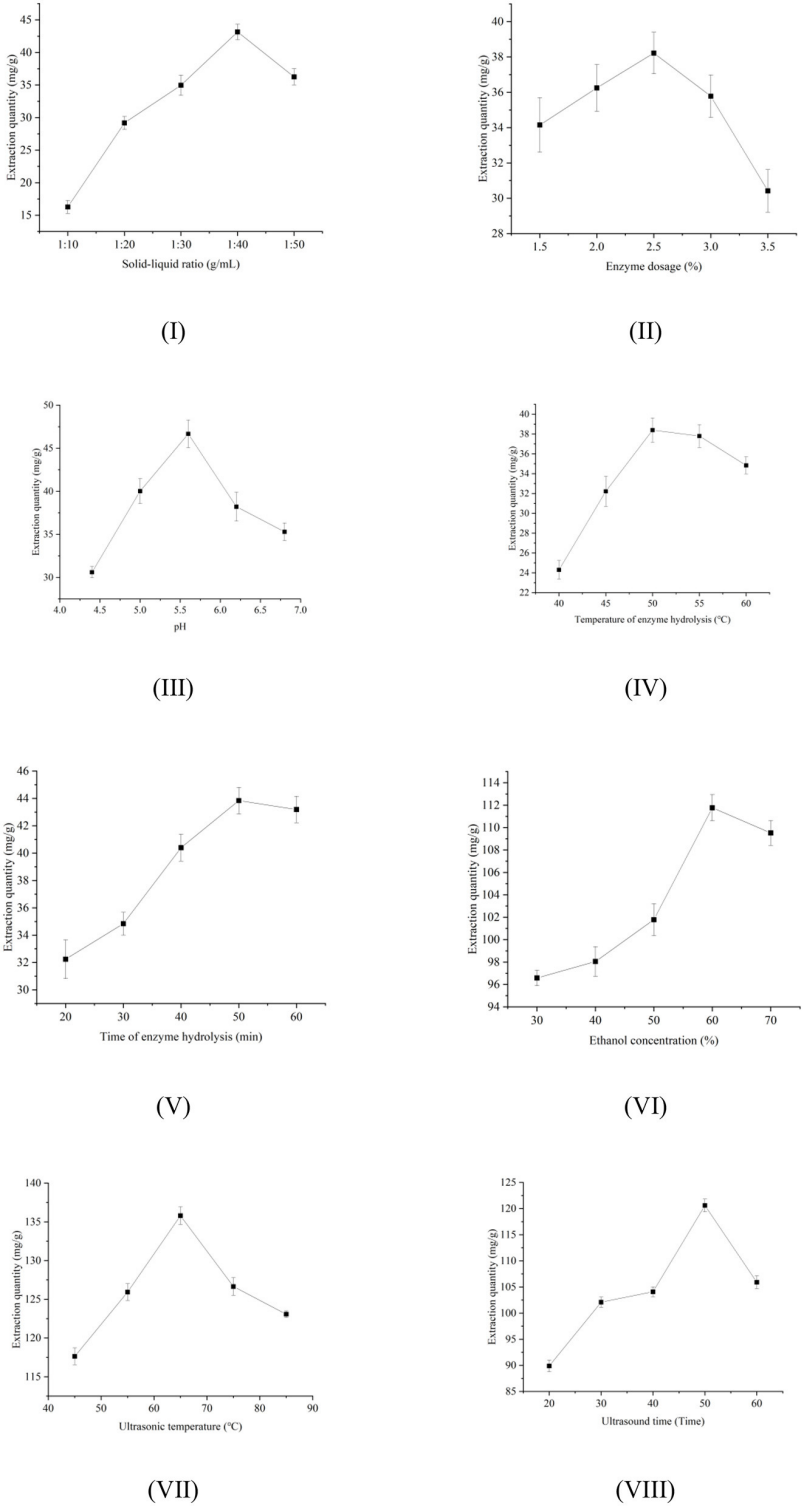
Effects of different factors on the efficiency of total polyphenols extraction. The influence of **(I)** solid-liquid ratio, **(II)** enzyme dosage, **(III)** pH, **(IV)** temperature of enzyme hydrolysis, **(V)** time of enzyme hydrolysis, **(VI)** ethanol concentration, **(VII)** ultrasonic temperature, and **(VIII)** ultrasonic time.

As shown in Figure 1I, as the solid: liquid ratio increases, the contact area between the dark tea powder and the ethanol solution increases, leading to the dissolution of total polyphenols and increased amounts of total polyphenols in the extracts. However, at ratios above 1:40, impurities are released from the tea leaves to compete with the total polyphenols for solvent, resulting in a decrease in the concentration of total polyphenols in the extract (22). As shown in Figure 1II, as the amount of cellulase increased, the degradation of the cell walls of the tea leaves increased, leading to greater release of the total polyphenols from the cells into the solution, and thus increased total polyphenols concentrations in the solution (23). However, when the concentration of cellulase exceeded 2.5%, the amount of extracted total polyphenols in the solution decreased, possibly due to the increased release of impurities from the cells, leading to reduced solubility of the total polyphenols (24). Figure 1III shows that the amount of extracted total polyphenols first increased and then decreased as the pH rose. The amount of extracted total polyphenols was greatest at pH 5.6, indicating that the activity of the enzyme was affected by pH, resulting in changes in conformation and dissociation status of the substrate (25). As shown in Figure 1IV, as the temperature of the enzyme hydrolysis increased, the thermal movement of molecules intensified, thereby enhancing the extraction yield of polyphenols and other bioactive compounds. However, at temperatures above 50°C, the enzyme has lost activity, and the polyphenol structures are also affected, thus reducing the amount of total polyphenols extracted (26). Figure 1V shows that longer hydrolysis times led to greater release of total polyphenols; however, when the duration of hydrolysis was longer than 50 min, phenolic acids can undergo oxidative decomposition, resulting in low total polyphenols concentrations in the extract (27). As shown in Figure 1VI, as the concentration of ethanol increased, the amount of total polyphenols in the extract first increased and then decreased. The highest amounts of extracted total polyphenols were seen at ethanol concentrations of 60%, which may be because solvents at certain concentrations can destroy hydrogen bonding and hydrophobic forces in phenols, polysaccharides, and proteins, increasing the extraction concentrations, but when the concentration is too high, the difference in polarity increases and thus the efficiency of extraction is reduced (28). In Figure 1VII, it can be seen that as the ultrasound temperature increased, the concentration of extracted total polyphenols first increased and then decreased, reaching its maximum at 65°C. The higher the ultrasonic temperature, the more favorable it is to the dissolution of total polyphenols, but if the temperature is too high, the ethanol will evaporate rapidly, and the increased temperature can easily lead to degradation of the polyphenol structure, thus adversely affecting the amount in the extract (29). As shown in Figure 1VIII, as the duration of ultrasonic treatment increased, the total polyphenols concentrations in the extract first increased and then decreased, with a peak at 50 min. Shorter periods of ultrasonication may be insufficiently effective for damaging the cell walls of the tea leaves, resulting in lower amounts of total polyphenols in the extract. Extending the time of ultrasonication will enhance cell wall breakdown and, thus, the release of total polyphenols from the cell interior. Times longer than 50 min can lead to greater exposure of the total polyphenols to adverse conditions, increasing the likelihood of oxidation and degradation, thus reducing the concentrations in the extract (30).

### 3.2 Plackett-Burman results

Using the results of the single-factor test and using the amount of extracted total polyphenols as the response variable, Plackett_Burman tests were performed for further identification of the optimal extraction conditions. The experimental design and results are shown in Table 5. Variance analysis and significance tests were performed on the test results, as shown in Table 6.

**Table 5:** Plackett-Burman experimental design and results.

**Number**	**Variable**								**Response value**
	**a**	**b**	**c**	**d**	**e**	**f**	**g**	**h**	**Extraction amount (mg/g)**
1	1:30	3	45	60	5.0	70	55	60	87 ± 1.18
2	1:50	3	45	40	6.2	50	55	60	99.69 ± 1.09
3	1:50	2	55	60	6.2	70	55	40	109.89 ± 1.08
4	1:30	2	45	40	6.2	70	75	40	94.41 ± 1.37
5	1:50	2	45	60	6.2	50	75	60	105.05 ± 1.61
6	1:30	2	55	60	5.0	50	75	60	92.96 ± 1.43
7	1:30	3	55	40	6.2	70	75	60	103.65 ± 1.20
8	1:50	3	55	40	5.0	50	75	40	105.61 ± 1.26
9	1:50	3	45	60	5.0	70	75	40	108.93 ± 1.37
10	1:30	3	55	60	6.2	50	55	40	86.3 ± 1.18
11	1:30	2	45	40	5.0	50	55	40	79.32 ± 1.15
12	1:50	2	55	40	5.0	70	55	60	112.17 ± 1.26

**Table 6:** Statistical analysis of the Plackett-Burman test.

**Source**	**Sum of squares**	**df**	**Mean squares**	***F*-value**	***P*-value**	**Significance**
Model	1,237.00	8	154.62	85.54	0.0019	^**^
a	795.44	1	795.44	440.06	0.0002	^**^
b	0.5720	1	0.5720	0.3165	0.6130	
c	1 09.08	1	109.08	60.35	0.0044	^**^
d	1.86	1	1.86	1.03	0.3855	
e	1 4.08	1	1 4.08	7.79	0.0683	
f	1 85.02	1	1 85.02	102.36	0.0021	^**^
g	1 09.44	1	109.44	60.55	0.0044	^**^
h	1 09.44	1	21.49	11.89	0.0410	^*^
Residual	5.42	3	1.81			

^**^The difference is highly significant (*P* < 0.01), ^*^The difference is significant (*P* < 0.05).

As can be seen from Table 6, the model is highly significant. The *P* value was < 0.01, and the determination coefficient *R*^2^ was 0.9956, indicating that the model was appropriate for 99.56% of the test data. The adjusted determination coefficient (Adjusted *R*^2^) was 0.9840, and the predicted determination coefficient (predicted *R*^2^) was 0.9302. The difference between the two was < 0.2, indicating that the model is reasonable. In terms of the significance of the experimental effect, the ranking of each factor was solid: liquid ratio > ethanol concentration > ultrasonic temperature > temperature of enzyme hydrolysis > ultrasonic time > pH > time of enzyme hydrolysis > enzyme dosage. Of these, the solid-liquid ratio (a), temperature of enzyme hydrolysis (c), ethanol concentration (f), and ultrasonic temperature (g) showed significant effects on the yield of tea total polyphenols. Therefore, in subsequent experiments, a cellulase concentration of 2.5% was used, together with a hydrolysis time of 50 min, pH of 5.6, and a 50 min duration of ultra-sonication. Four factors, namely, the solid: liquid ratio, temperature of enzyme hydrolysis, ethanol volume fraction, and ultrasonic temperature, were selected for further investigation. The effects of these four factors and their interactions on the efficiency of total polyphenols extraction were then analyzed.

### 3.3 Experimental design of response surface optimization

After determining the key factors of dark tea total polyphenols extraction using the Plackett-Burman test, Design Expert 13 was used for the inclusion of the four independent variables into a total of 27 sets of tests with 4 factors and 3 levels. The results of the response surface experiment are shown in Table 7. The total polyphenols concentration in the extract (Y) was used as the response value to perform regression fitting with each factor. The quadratic polynomial regression equation was obtained as: 150.86 + 1.99A + 11.35B + 0.9708C + 4.7D – 8.89AB + 5.93AC + 3.75AD – 4.60BC + 3.97BD – 3.29CD – 5.15A^2^ – 0.4321B^2^ + 3.34C2 – 6.25D^2^.

**Table 7:** Box-Behnken experimental design and results.

**Run**	**A**	**B**	**C**	**D**	**Extraction amount (mg/g)**
1	50	50	70	65	158.89 ± 1.29
2	50	30	60	55	131.21 ± 1.51
3	55	40	70	65	156.28 ± 1.69
4	45	40	70	65	142.13 ± 1.49
5	50	50	60	75	164.75 ± 1.42
6	50	30	70	65	150.12 ± 1.59
7	45	40	50	65	153.33 ± 1.54
8	50	50	60	55	146.76 ± 1.22
9	45	30	60	65	120.43 ± 1.12
10	45	40	60	75	136.23 ± 1.44
11	50	30	50	65	140.01 ± 1.65
12	50	40	50	55	135.87 ± 1.19
13	50	50	50	65	167.17 ± 1.53
14	50	40	60	65	150.33 ± 1.37
15	55	30	60	65	143.24 ± 1.39
16	55	40	50	65	143.78 ± 1.58
17	45	40	60	55	138.66 ± 1.11
18	50	40	70	75	153.21 ± 1.31
19	45	50	60	65	164.84 ± 1.11
20	55	40	60	75	148.32 ± 1.41
21	50	40	70	55	146.71 ± 1.22
22	55	40	60	55	135.74 ± 1.67
23	50	40	50	75	155.53 ± 1.47
24	50	30	60	75	133.34 ± 1.44
25	50	40	60	65	153.54 ± 1.45
26	50	40	60	65	158.89 ± 1.77
27	55	50	60	65	131.21 ± 1.58

It can be seen from Tables 8, 9 that the *P*-value of the model (*P* < 0.0001) indicates that the model is extremely significant and has reference value and research significance. The lack of fit of the model *P*-value was 0.4871, which was not significant, showing that the model fully fitted the experimental data with low error and minimal effects of unknown factors. The model equation *R*^2^ = 0.9697 indicated that the regression model fitted well and that the regression equation was representative and could predict the actual situation more accurately. The adjusted determination coefficient $R_{\text{adj}}^{2}$ was 0.9343, indicating that only 6.57% of the total variation could not be explained by the model. The Adeq precision is the ratio of detecting noise signal, which is usually greater than 4. In this experiment, it was found to be 21.5935, indicating that the model was a true reflection of the experimental results. The CV value indicates the accuracy of the experiment. The lower the value, the higher the reliability of the experiment. In this experiment, the value was found to be 1.93%, indicating that the model has high accuracy and can be applied to optimize the extraction process of dark tea total polyphenols. The order of factors affecting the extraction amount of dark tea total polyphenols is solid: liquid ratio (B) > ultrasonic temperature (D) > temperature of enzyme hydrolysis (A) > ethanol concentration (C). The solid-liquid ratio (B), ultrasonic temperature (D), interaction term (AB), interaction term (BC), temperature of enzyme hydrolysis quadratic term (A^2^), and ultrasonic temperature quadratic term (D^2^) all reached the level of extreme significance.

**Table 8:** Analysis of variance of the regression equation for the Box-Behnken test.

**Source**	**Sum of squares**	**df**	**Mean square**	***F*-value**	***P*-value**	**Significance**
Model	3,091.86	14	220.85	27.41	< 0.0001	^**^
A	47.40	1	47.40	5.88	0.0320	^*^
B	1,545.19	1	1545.19	191.77	< 0.0001	^**^
C	11.31	1	11.31	1.40	0.2590	
D	265.36	1	265.36	32.93	< 0.0001	^**^
AB	315.77	1	315.77	39.19	< 0.0001	^**^
AC	140.42	1	140.42	17.43	0.0013	^**^
AD	56.33	1	56.33	6.99	0.0214	^*^
BC	84.55	1	84.55	10.49	0.0071	^**^
BD	62.88	1	62.88	7.80	0.0162	^*^
CD	43.30	1	43.30	5.37	0.0389	^*^
A^2^	141.71	1	141.71	17.59	0.0012	^**^
B^2^	0.9957	1	0.9957	0.1236	0.7313	
C^2^	59.42	1	59.42	7.37	0.0188	^*^
D^2^	208.31	1	208.31	25.85	0.0003	^**^
Residual	96.69	12	8.06			
Lack of fit	84.60	10	8.46	1.40	0.4871	Not significant
Pure error	12.09	2	6.04			
Cor total	3,188.55	26				

^**^Means highly significant (*P* < 0.01), ^*^Means significant (*P* < 0.05).

**Table 9:** Reliability analysis of regression model.

**Source**		**Source**	
Std. Dev.	2.84	*R* ^2^	0.9697
Mean	147.08	Adjusted r^2^	0.9343
C.V. %	1.93	Predicted *R*^2^	0.8386
		Adeq precision	21.5935

**Figure 2:**
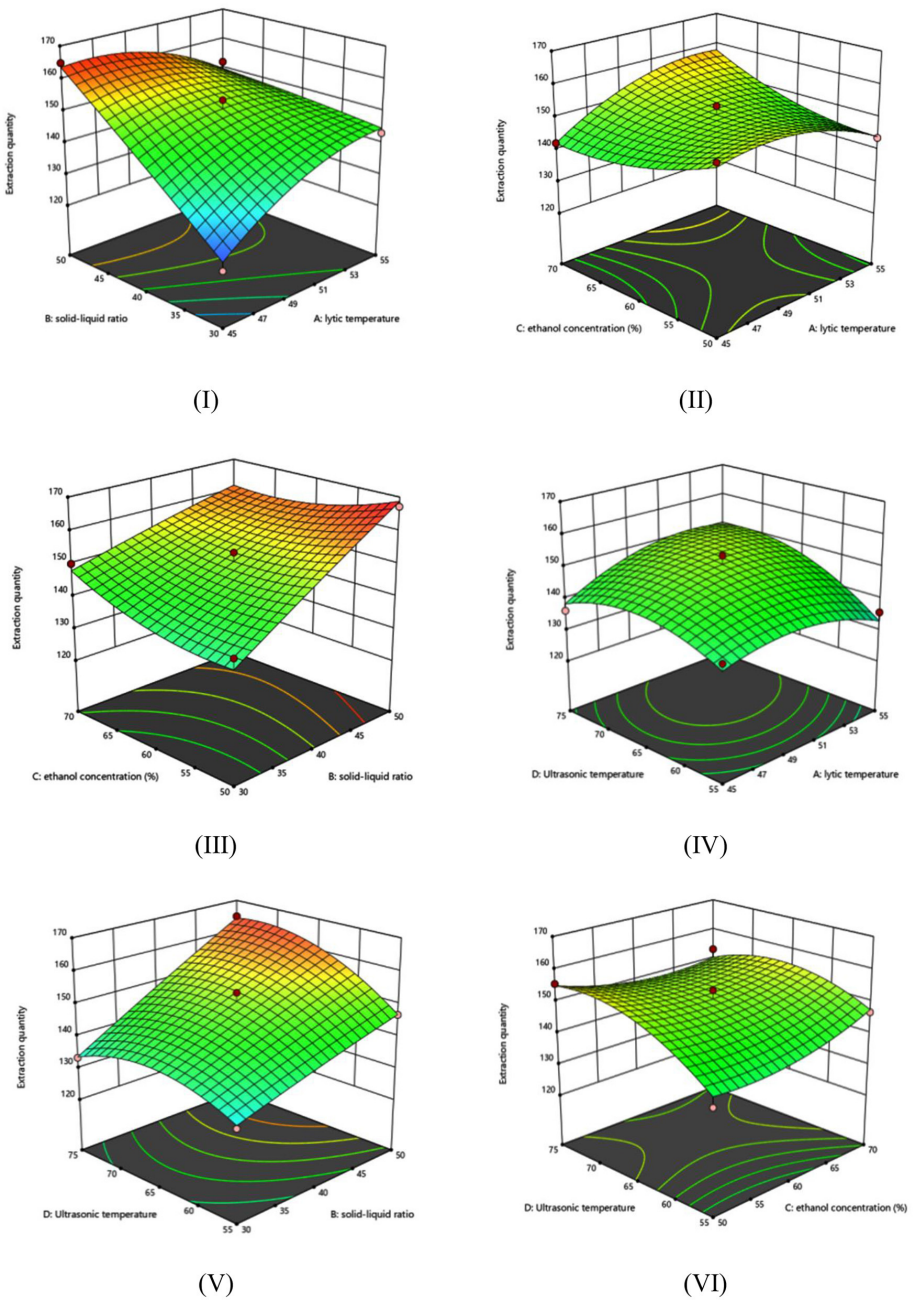
Response surface diagram of the interaction of various experimental factors. **(I)** Interaction between solid-liquid ratio and lytic temperature, **(II)** interaction between ethanol concentration and lytic temperature, **(III)** interaction between ethanol concentration and solid-liquid ratio, **(IV)** interaction between ultrasonic time and lytic temperature, **(V)** interaction between ultrasonic time and solid-liquid ratio, and **(VI)** interaction between ultrasonic temperature and ethanol concentration.

The effect of each factor on the response value is shown in Figures 2I–VI. The AB, AC, and BC terms showed high color variation, high slope degrees, and steep curves, indicative of extremely significant interactions. AD, BD, and CD showed large color variations and high slope degrees, indicating significant interaction. This is consistent with the results of the variance analysis.

### 3.4 Response surface result verification

The optimal extraction scheme for dark tea total polyphenols predicted by the software and the actual measurement results are shown in Table 10.

**Table 10:** Verification of response surface results.

**Category**	**A**	**B**	**C**	**D**	**Extraction amount (mg/g)**
Predicted value	45.0017	1:50	50.0001	71.6748	179.1 ± 0.13
Actual value	45	1:50	50	72	178.65 ± 0.36

After analysis, the difference between the predicted and the actual results was only 0.45 mg/g, demonstrating the accuracy and reliability of the model in the prediction of total polyphenols extraction.

### 3.5 Effects of different extraction methods on the efficiency of total polyphenols extraction

Table 11 shows the effects of three different extraction methods on the extraction amount of dark tea total polyphenols. The order of dark tea total polyphenols extraction amount from high to low is ultrasound assisted enzymatic method>ultrasound assisted extraction method > ethanol reflux method. The ultrasonic assisted extraction method increased the extraction amount of dark tea total polyphenols by about 18.58%, shortened the extraction time by 37.5%, and reduced the extraction temperature by 13.75% compared to the ethanol reflux extraction method. The reason may be that the ultrasonic generated cavitation effect destroyed the structure of the raw material cell wall, resulting in a larger contact area between the solid-liquid phase and easier dissolution of the active ingredients (31). The ultrasonic assisted extraction method increased the extraction amount of dark tea total polyphenols, reduced the extraction time, and lowered the extraction temperature. The ultrasonic assisted enzymatic method increased the extraction yield of dark tea total polyphenols by about 43.38% compared to the ethanol reflux method; Compared with using ultrasound assisted extraction alone, the amount of dark tea total polyphenols extracted by cellulase hydrolysis followed by ultrasound extraction significantly increased, by about 30.45%. The plant cell wall is a complex network structure composed of cellulose, hemicellulose, pectin, and a small amount of structural proteins. In the cell wall structure rope network model, cellulose crystals are connected to hemicellulose through hydrogen bonds, forming a network of fibers and hemicellulose. Hydrophilic pectin and a small amount of structural proteins fill the gaps in the network structure (32). With cellulase hydrolysis, the cell wall of dark tea breaks down, promoting the continuous release of phenolic components trapped in the cell wall and increasing the extraction amount of phenolic components. The use of cellulase can first break down substances such as cellulose and hemicellulose in the cell wall and interstitium, disrupting the dense structure of the cell wall. Based on this, the cavitation and microenvironment stirring effects of ultrasound further promote the dissolution of active ingredients in the cell (33). Therefore, the ultrasound assisted enzymatic extraction method for dark tea total polyphenols is simple, efficient, low-cost, environmentally friendly, and can be industrially promoted. It also has reference value for the extraction of other plant active ingredients.

**Table 11:** Effects of different extraction methods on the amount of extracted dark tea total polyphenols.

**Items**	**Extraction methods**		
	**G**	**H**	**I**
Extract solvent	50% ethanol	50% ethanol	50% ethanol
Extraction time (min)	80	50	50
Extraction temperature (°C)	80	72	72
Solid-liquid ratio (ml/g)	50:1	50:1	50:1
Ultrasonic power (W)	-	360	360
Time of enzymatic hydrolysis (min)	-	-	50
pH	-	-	5.6
Temperature of enzyme hydrolysis (°C)	-	-	45
Enzyme quantity (%)	-	-	2.5
Extraction amount (mg/g)	101.052 ± 0.19	124.112 ± 0.87	178.46 ± 0.98

### 3.6 Antioxidant activity of dark tea total polyphenols extracted by different methods

**Figure 3:**
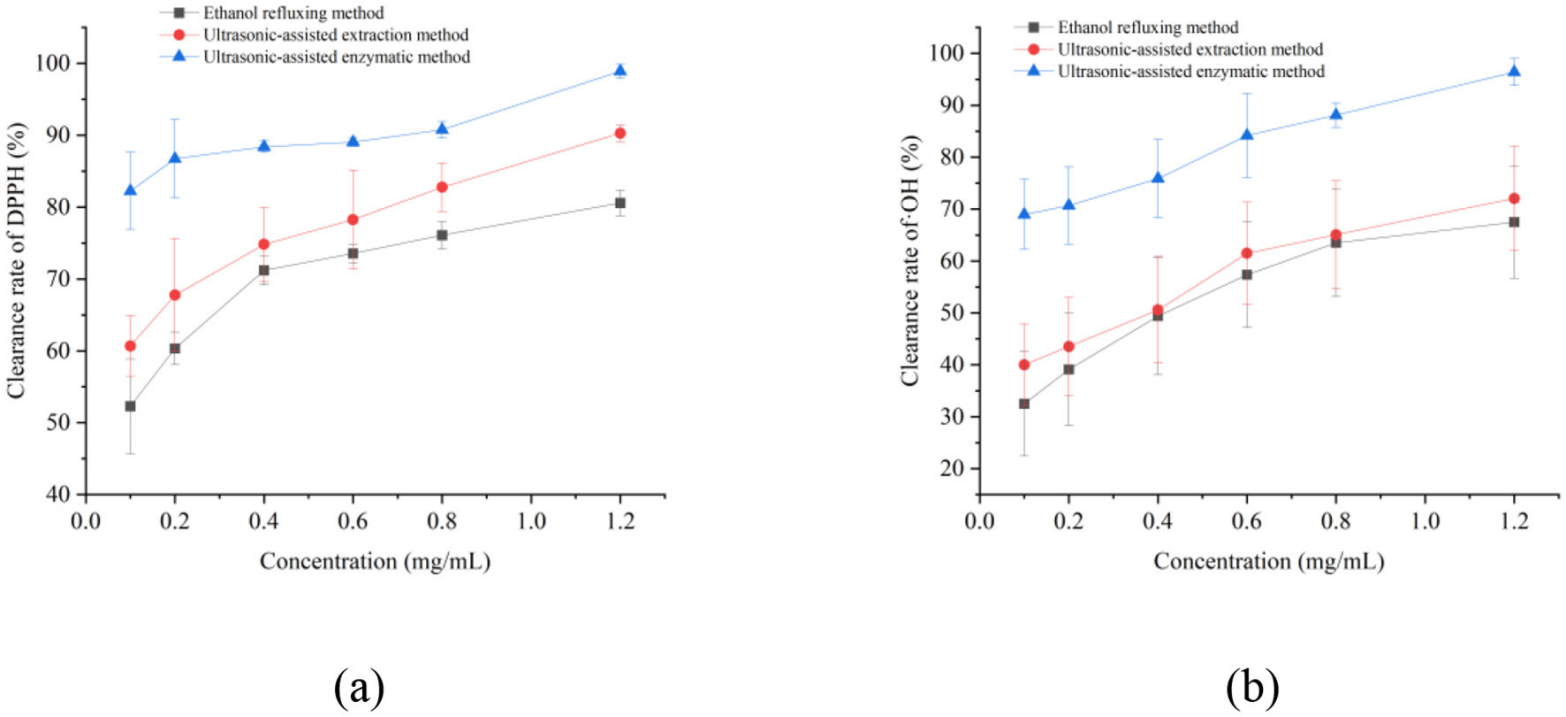
Clearance of DPPH and ·OH free radicals by different concentrations of total polyphenols. **(a)** DPPH radical scavenging ability and **(b)** ·OH radical scavenging ability.

The antioxidant activity results of dark tea total polyphenols prepared by different extraction methods are shown in Figure 3. As the concentration increases, the ability of dark tea total polyphenols extracted by different methods to scavenge DPPH radicals (Figure 3A) and · OH radicals (Figure 3B) shows a concentration dependent relationship. Under the same concentration, dark tea total polyphenols extracted by ultrasound assisted enzymatic method have the strongest ability to scavenge DPPH radicals and OH radicals, followed by ultrasound assisted extraction method, and the ethanol reflux method has the lowest efficacy. This is consistent with the conclusion that the ultrasound assisted enzymatic extraction method obtained the highest content of dark tea total polyphenols in the aforementioned antioxidant activity experiment. The study has shown (34–36) that the bioactivity of polyphenols is closely related to their composition and content. Due to the significant differences in the composition and content of polyphenols prepared by different extraction methods, the bioactivity of polyphenols in dark tea also varies significantly depending on the extraction method used (37, 38). The type and origin of the herbal medicine, the composition and content of polyphenols, as well as the methods of extraction and isolation, may all influence their bioactivity (39–41).

### 3.7 Correlation analysis between total polyphenols content and free radical scavenging ability using different extraction methods

The Pearson correlation between total polyphenols content and free radical scavenging ability obtained by different extraction methods is shown in Table 12. The total polyphenols content obtained by different extraction methods is significantly positively correlated with DPPH·, ·OH free radical scavenging rate (*P* < 0.01). The correlation coefficients between dark tea total polyphenols content and DPPH· scavenging rate obtained by ethanol reflux extraction, ultrasound assisted extraction, and ultrasound assisted enzyme method are 0.890, 0.877, and 0.850, respectively. The correlation coefficients between dark tea total polyphenols content and ·OH free radical scavenging rate obtained by ethanol reflux extraction, ultrasound assisted extraction, and ultrasound assisted enzyme method are 0.786, 0.807, and 0.880, respectively, indicating that the higher the total polyphenols content, the higher the DPPH·, ·OH free radical scavenging rate, indicating that polyphenolic compounds are dark tea. It is evident that polyphenolic compounds are key functional components in the scavenging of DPPH· and ·OH radicals in dark tea. Due to variations in extraction methods, the content of polyphenols extracted differs, and consequently, their ability to eliminate free radicals also varies (42, 43).

**Table 12:** Correlation analysis between dark tea total polyphenols content and DPPH and ·OH scavenging rates using different extraction methods.

**Items**	**Clearance rate of DPPH**			**Clearance rate of** ·**OH**		
**Dark tea total polyphenols content**	**G**	**H**	**I**	**G**	**H**	**I**
Correlation coefficient/*R*^2^	0.890^**^	0.877^**^	0.850^**^	0.786^**^	0.807^**^	0.880^**^
*P* value	< 0.001	< 0.001	< 0.001	< 0.001	< 0.001	< 0.001
Sample size	18	18	18	18	18	18

^**^At the 0.001 level (double tailed), the correlation is significant.

### 3.8 Composition and content of total polyphenols in dark tea extracts by different extraction methods

**Figure 4:**
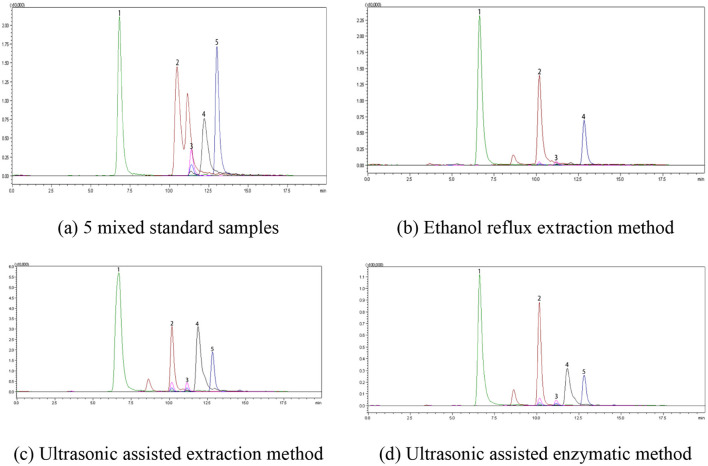
LC-MS/MS chromatogram. **(a)** 5 mixed standard samples, **(b)** ethanol reflux extraction method, **(c)** ultrasonic assisted extraction method, and **(d)** ultrasonic assisted enzymatic method.

**Figure 5:**
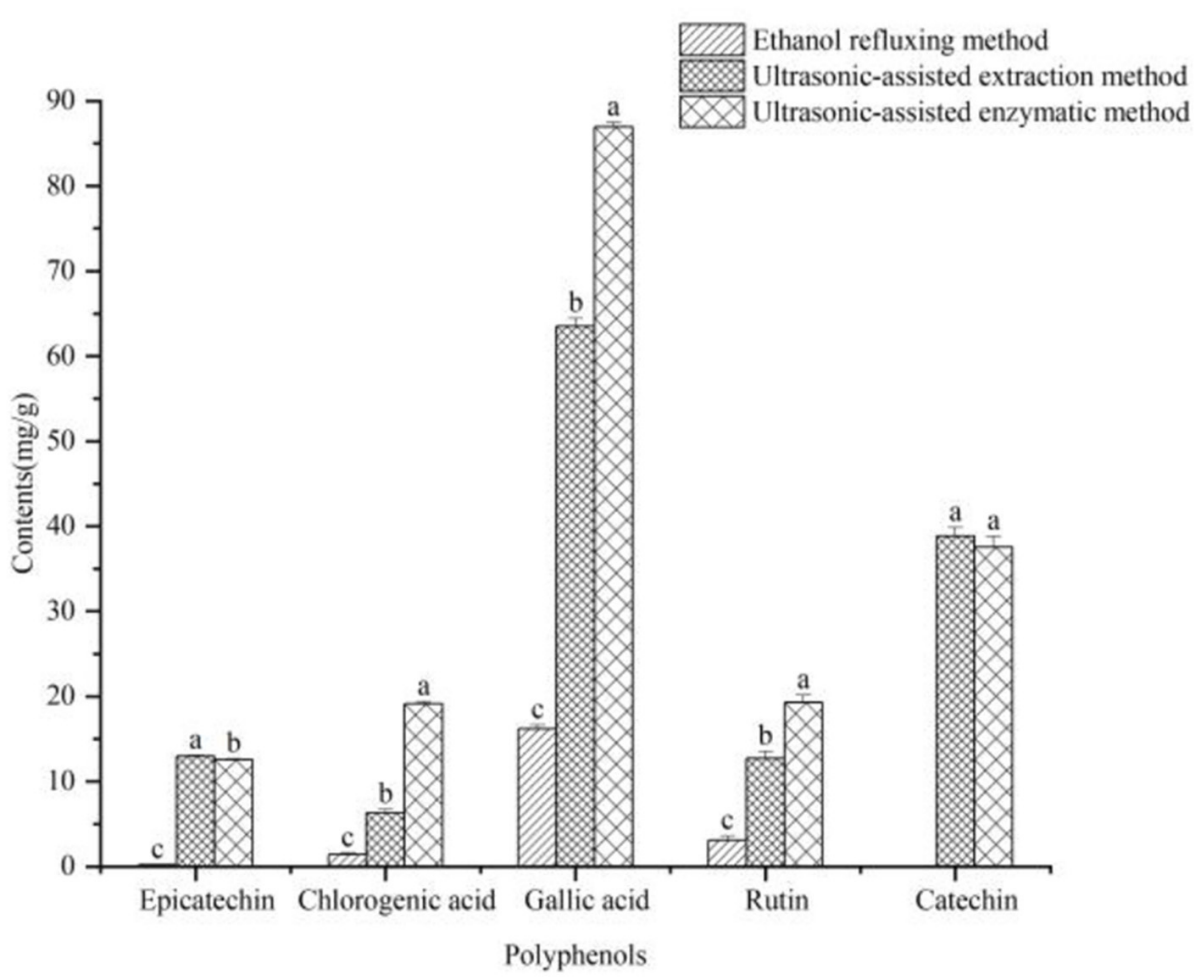
shows the total polyphenols content (epicatechin, chlorogenic acid, gallic acid, rutin, catechin) in dark tea extracts obtained by ethanol reflux extraction, ultrasound assisted extraction, and ultrasound assisted enzymatic extraction; Each group was repeated three times, with different lowercase letters representing significant differences (*P* < 0.05).

According to LC-MS/MS analysis (Figure 4), five polyphenolic components including epicatechin, chlorogenic acid, gallic acid, rutin, and catechin were detected in dark tea extracts extracted by different extraction methods; Qualitative analysis of these five total polyphenols in dark tea extracts extracted by different extraction methods was conducted by retention time and relative abundance of ions, and quantitative analysis of the five total polyphenols in dark tea extracts extracted by different extraction methods was performed using external standard method. The content of total polyphenols in dark tea extracts extracted by different extraction methods is shown in Figure 5. The results revealed that Gallic acid (169.16 ± 1.62 mg/g) is the most abundant phenolic compound, followed by catechins (79.42 ± 1.18 mg/g), rutin (33.22 ± 1.36 mg/g), chlorogenic acid **(**28.14 ± 0.85 mg/g**)**, and epicatechin (26.84 ± 0.97 mg/g). These compounds are the main contributors to the antioxidant activity of dark tea extract. In addition, the total polyphenols content extracted by ultrasound assisted enzymatic method was the highes, and there was a significant difference (*P* < 0.05) between the total polyphenols content extracted by ultrasound assisted extraction method and ethanol reflux extraction method. This is consistent with previously reported findings (44–47), which suggest that ultrasonic-assisted enzyme methods can significantly enhance both the extraction yield and antioxidant activity of polyphenols in dark tea.

## 4 Conclusion

This study constructed a response surface model for the ultrasound-assisted enzymatic extraction of dark tea total polyphenols using single-factor, Plackett-Burman, and Box-Behnken experiments and determined the optimal extraction conditions to be a pH of 5.6, time of enzymatic hydrolysis of 50 min, a temperature of enzymatic hydrolysis of 45°C, a solid: liquid ratio of 1:50, an ethanol volume fraction of 50%, an ultrasound temperature of 72°C, ultrasound time of 50 min, and enzyme concentration of 2.5%. Under these conditions, the amount of dark tea total polyphenols extracted was 178.65 mg/g, which was not significantly different from the value predicted by the model (179.1 mg/g) (*P* > 0.05). The total polyphenols content obtained by different extraction methods is significantly correlated with antioxidant activity. The optimized process of ultrasound assisted enzymatic extraction can increase the extraction amount and antioxidant activity of dark tea total polyphenols, reduce extraction time, and lower extraction temperature. Ultrasonic assisted enzymatic method is simple, efficient, and can be industrially promoted, and it also has reference value for the extraction of other plant active ingredients.

## Data Availability

The original contributions presented in the study are included in the article/supplementary material, further inquiries can be directed to the corresponding authors.
